# Retention of knowledge and perceived relevance of basic sciences in an integrated case-based learning (CBL) curriculum

**DOI:** 10.1186/1472-6920-13-139

**Published:** 2013-10-08

**Authors:** Bunmi S Malau-Aduli, Adrian YS Lee, Nick Cooling, Marianne Catchpole, Matthew Jose, Richard Turner

**Affiliations:** 1 School of Medicine, University of Tasmania, Hobart, Australia

## Abstract

**Background:**

Knowledge and understanding of basic biomedical sciences remain essential to medical practice, particularly when faced with the continual advancement of diagnostic and therapeutic modalities. Evidence suggests, however, that retention tends to atrophy across the span of an average medical course and into the early postgraduate years, as preoccupation with clinical medicine predominates. We postulated that perceived relevance demonstrated through applicability to clinical situations may assist in retention of basic science knowledge.

**Methods:**

To test this hypothesis in our own medical student cohort, we administered a paper-based 50 MCQ assessment to a sample of students from Years 2 through 5. Covariates pertaining to demographics, prior educational experience, and the perceived clinical relevance of each question were also collected.

**Results:**

A total of 232 students (Years 2–5, response rate 50%) undertook the assessment task. This sample had comparable demographic and performance characteristics to the whole medical school cohort. In general, discipline-specific and overall scores were better for students in the latter years of the course compared to those in Year 2; male students and domestic students tended to perform better than their respective counterparts in certain disciplines. In the clinical years, perceived clinical relevance was significantly and positively correlated with item performance.

**Conclusions:**

This study suggests that perceived clinical relevance is a contributing factor to the retention of basic science knowledge and behoves curriculum planners to make clinical relevance a more explicit component of applied science teaching throughout the medical course.

## Background

The current paradigm of medical education has been called into question as the demands for the quality and quantity of medical graduates increases [1]. The optimal integration of basic medical sciences into undergraduate education and then, eventually, into clinical practice is one such area under scrutiny [2].

For medical students to make competent clinical decisions based on sound scientific principles, they must be able to retain knowledge from the preclinical phase of their medical course [3-6]. Evidence from published literature indicates that failure rates on certifying examinations and board certification status were significantly associated with the assessment of retained basic sciences knowledge from medical school education [7]. D’Eon [8] found a considerable knowledge loss among medical students in immunology (13%), neuro-anatomy (46.5%) and physiology (16%) on retest ten months later. He concluded that knowledge loss did not seem to be related to the marks on the final examination or the assessment of course quality by the students. Ling et al. [9] revealed dramatic decline in examinee performance in biochemistry, followed by microbiology and pharmacology during the Step 1 United States Medical Licensing Examination (USMLE) in comparison to the Step 2 USMLE. Observed gains in physiology, anatomy and pathology appeared to be related to reinforcement of material during patient care in clerkships. Harris et al. [10] concluded that when knowledge gained is not directly relevant or applicable to clinical contexts, it is lost rather quickly. They recommended that in order to prevent extensive loss of knowledge, the information given must be relevant. Perceived relevance of a subject matter facilitates knowledge retention and application, while a lack of relevance is associated with the converse of this [11].

The Australian Medical Study (AMES) [12] in 2008 evaluated the critical educational factors contributing to the outcomes of undergraduate medical education in Australia. They reported variation across Australia in the amount, type and method of bioscience teaching and learning within medical school programs. Studies have also shown that demographic factors such as student gender, age, language background and ethnicity influence retention and academic performance [13-15]. Some studies have reported higher academic achievements by female students and more mature students in comparison to their male and younger counterparts respectively [13,15]. However, there are inconsistent results on the relationship between these factors and academic success [14]. Furthermore, their influence on the retention of basic science knowledge needs to be further explored.

### Study context

The Tasmanian School of Medicine (TSoM) has a five-year undergraduate entry Case-Based Learning (CBL) curriculum which includes vertical and horizontal integration of basic sciences and clinical teaching. Vertical integration of the curriculum is promoted through a thematic structure usage in all the five years of the program. Students are increasingly (from Years 1 to 5) exposed to clinical situations through CBL tutorials and clinical placements. The first two years of the course provide a systems-based introduction to the foundations of medicine, with an early opportunity to develop communication and clinical skills. In the third year of the program, students commence clinical rotations in Medicine, Surgery, Primary Care and specialty areas (Obstetrics & Gynaecology, Psychiatry and Paediatrics); whilst the final two clinical years are taught at one of three dedicated clinical schools where learning is consolidated in the context of clinical and community placements.

The value of CBL lies in exploiting the “basic human capacity to learn from stories” [16]. CBL has been acknowledged as a structured approach to collaborative learning that consolidates and integrates newly acquired knowledge and skills [17,18]. CBL ensures the consolidation of newly acquired knowledge through its application and discussions [18,19]. With this integrated approach, the learner is required to gather information, prioritise according to relevance or importance and filter out irrelevant information [20]. We therefore hypothesise that the demonstrated relevance and applicability of basic sciences to clinical situations will foster retention of gained knowledge. Using a cross-sectional study design, we aimed to answer the following research questions:

•What are the demographic factors that influence retention of basic science knowledge?

•Does perceived clinical relevance of basic science materials relate to retention of knowledge?

## Methods

### Participants

All Years 2 to 5 medical students (approximately 120 students per class) were invited to participate in this study. Year 1 students were excluded from the study because it was considered unlikely that they would have had sufficient course time to integrate basic science into clinical contexts and make informed judgments on the clinical relevance of the assessment items. Face-to-face cohort announcements were made to encourage participation, and as an incentive, participants were given the opportunity to enter into a drawing to win an iPad. Students were assured of no adverse academic repercussions for non-participation. The study was conducted in September 2012, two (2) months prior to the students’ end of year examinations. This was to avoid the confounding effect of students studying for their summative exams. Feedback on their total scores and summary statistics was provided, three (3) weeks after the exams, to participants who elected to receive it. Ethics approval for this study was obtained from the Tasmanian Social Sciences Human Research Ethics Committee [HREC project number: H0012584].

### Procedure

Students were asked to complete an 80-minute paper-based examination consisting of fifty (50) A-type (single best response of five) multiple-choice questions (MCQs) from past second year written examinations in a lecture theatre setting. This formative assessment tested student knowledge on five (5) basic science disciplines with equal weightings. These were anatomy, physiology, pharmacology, pathology (including microbiology and immunology) and biochemistry. Ten (10) MCQs were used for each discipline. At the start of the examination, participants were instructed to provide demographic information including: identification number, year of study, age, gender, student origin, highest level of previous qualification, proposed future career and if they were involved in academic tutoring.

Examination questions with difficulty index of 0.3-0.8 (defined as the proportion of previous students who answered the question correctly) and discrimination index of 0.3-1.0 (the difference between the proportions of the high and low performing groups) were randomly selected from our existing validated item bank [21] using the IDEAL software package [22]. The examination questions had been pre-validated through blueprinting (the mapping of test items to the intended learning outcomes) and item analysis (statistical process that assesses the quality of test items). Due to the random selection, not all questions had clinical vignettes. The students were instructed to answer all questions to the best of their ability and then rate each question on a Likert scale of 1–5 according to their perception of its relevance to the clinical practice of medicine (with 1 = highly irrelevant and 5 = highly relevant). For the purpose of this study, clinical relevance is defined as the practical applicability of the basic science item to the hospital setting. Care was taken to explain to the participants that the standard was targeted at an intern/junior doctor level.

### Statistical analyses

A power analysis [23] calculation was conducted prior to commencement of study to determine sample size. A General Linear Model (GLM) procedure in SAS [24] was utilised in computing least square means of the explanatory variables, their effect on participants’ test scores as well as differences in retention of knowledge in individual disciplines. The initial model included the fixed effects of sex, gender, student origin, year of study, involvement in tutoring, highest level of previous qualification and proposed future career. Only significant factors and their two-way interactions were retained in the final model. Significant levels were set at p < 0.01. Effect sizes were calculated using partial eta-squared (η_p_^2^), to determine the magnitude of statistically significant relationships. The effects were interpreted as defined by Cohen [25]: small effect - >0.01; medium effect - >0.058, and large effect - >0.137. Pearson correlation coefficients between variables were computed to establish the strength and direction of associations; and significance was tested using Bonferroni probability. To enable comparison, all scores were expressed as percentages of the correct answers in the total test and individual disciplines. In addition, for each item in the examination, a ratio using the percent score achieved by the participants divided by the average score achieved on the same item when it was administered to the 'reference group’ – a cohort of Year 2 (preclinical) students, was computed. This ratio, which is a possible indicator of knowledge retention, was correlated with perceived relevance [10]. Reproducibility (Kuder-Richardson 20 - KR-20) of the test was calculated using the classical test theory as provided in IDEAL 4.1, an Item Analysis Program [26]. To ensure that participants were representative of their respective classes, their mean scores on regular in-course formative and summative examinations were compared with those of their non-participating counterparts. This was calculated using a two-tailed *t* test.

## Results

Two hundred and thirty-two (232) students participated in this study, representing 50.0% of the Years 2–5 students. This comprised 44 Year 2 students (40.7% response rate), 71 Year 3 students (60.7%), 62 Year 4 students (50.4%) and 55 Year 5 students (47.4%). The mean age of the study group was 22.7 ± 3.38 years (range 18–49 years). The cohort comprised of 78.9% domestic (n = 183) and 21.1% international (n = 49) students and there were more females than males (56.0% (n = 130) vs 44.0% (n = 102)). Most of the participants (59.1%, n = 137) were undecided about their future specialty areas; only 22.0% of them (n = 51) were involved in tutoring and only 12.9% of them (n = 30) had previously completed a tertiary degree. In addition, comparison of participants’ in-course examination results with those of their non-participating colleagues were non-significant, indicating study participants were representative of their respective cohorts (see Table 1).

**Table 1:** Comparison of performance between participants and non-participants in previous exams

**Year of study**	**Participants (mean ± SD)**	**Non-participants (mean ± SD)**	***T*-Test**
Year 2	56.2 ± 11.7	55.0 ± 10.6	0.56
Year 3	69.0 ± 8.0	66.0 ± 6.4	0.20
Year 4	59.1 ± 8.4	57.0 ± 11.0	0.29
Year 5	70.6 ± 7.1	70.5 ± 6.7	0.70

**Figure 1:**
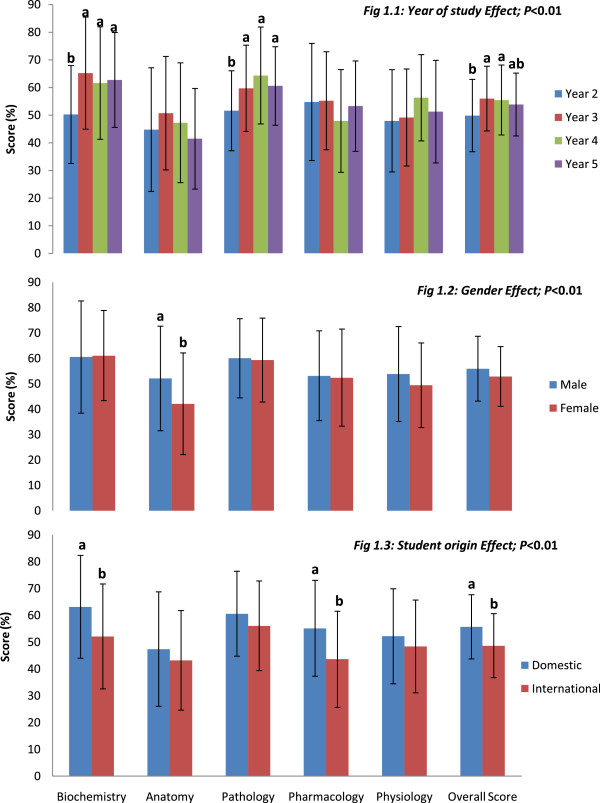
**Mean student scores (± standard deviation) and level of significance (P-values) for each discipline and overall exam score.** Within discipline, differences are signified by letters above columns, differing superscripts **(a, b)** show significant differences (P < 0.01).

On the whole, Year 2 students had significantly lower overall exam scores than the Years 3 and 4 but not Year 5. The mean score for the examination was 54.18% (SD = 6.13; range = 26-84%). The KR-20 reliability was 0.72 and the standard error of measurement (SEM) was 3.26. As shown in Figure 1, significant effects (p < 0.01) were seen on mean discipline-specific and overall scores, according to year of study, gender and origin of the participants. Although Year 2 students had the least scores in all domains, the difference was only significant for biochemistry (p = 0.0002; η_p_^2^ = 0.10), pathology (p = 0.007; η_p_^2^ = 0.07) and the overall exam score (p = 0.009; η_p_^2^ = 0.06) (Figure 1). Irrespective of year of study, similar discipline-specific performance trends were observed, with each cohort obtaining lowest scores in anatomy (Figure 1.1). As shown in Figure 1.2, the male students outperformed the females, with significantly higher scores in anatomy (p = 0.002; η_p_^2^ = 0.05). Domestic students had significantly higher scores than international students in biochemistry (p = 0.001; η_p_^2^ = 0.06), pharmacology (p < 0.0001; η_p_^2^ = 0.08) and the overall exam scores (p = 0.0004; η_p_^2^ = 0.07). Mature-aged (>25 years) participants and those who had been involved in tutoring obtained higher scores than their counterparts respectively, but the differences were not significant (see Additional file 1). No significant differences in performances were seen for students who had previously completed a tertiary degree.

Significant (p < 0.05) origin by age-group interaction effect was observed in anatomy and physiology where younger domestic students obtained higher scores than their older counterparts, while the reverse was the case for international students. Sex by age-group interaction effects were also significant (p < 0.05) for biochemistry, anatomy and overall scores where younger male students outperformed older male students. Conversely, older female students did better than the younger ones. Significant (p < 0.01) year of study by sex interactions were observed in the pathology and pharmacology scores. In pathology, Years 3 and 5 male students performed better than their female counterparts; while in pharmacology, Years 4 and 5 males performed better than the females.

**Figure 2:**
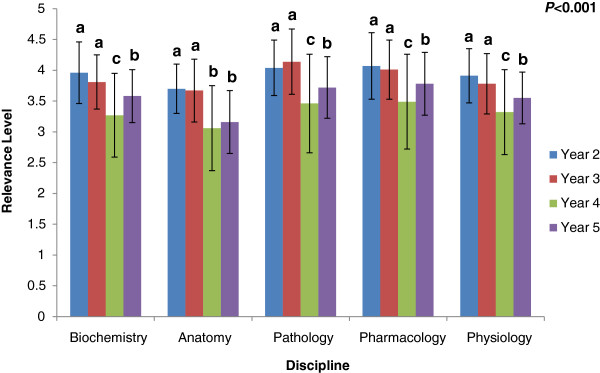
**Mean clinical relevance ratings (± standard deviation) by Year of study and level of significance (*P*-values) for each subject and overall exam score.** Within discipline, differences are signified by letters above columns, differing superscripts **(a, b, c)** show significant differences (*P* < 0.01).

**Figure 3:**
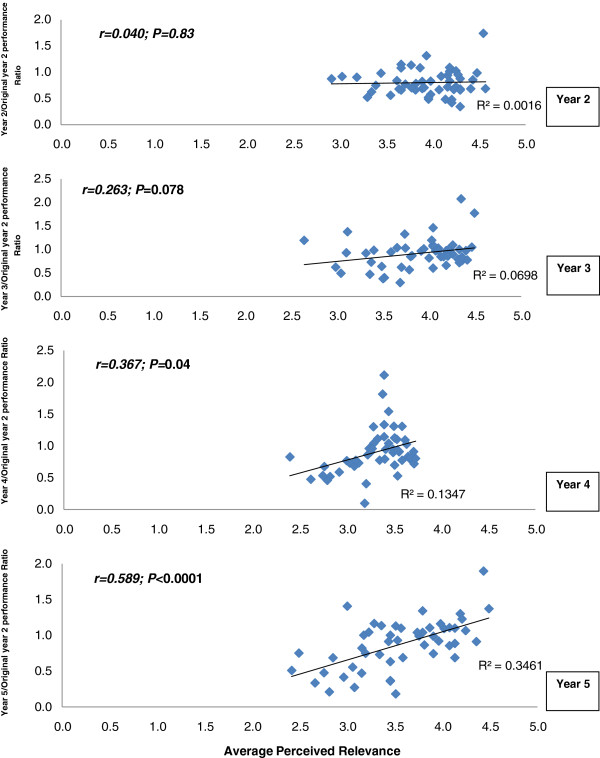
Correlation of perceived relevance with knowledge retention ratio for each year group, including r and p-values.

In comparison to their senior colleagues, Years 2 and 3 students gave significantly higher (p < 0.001) ratings of the clinical relevance of all the basic science subjects. In general, Year 4 students consistently gave the lowest ratings (see Figure 2). Figure 3 depicts the correlation between perceived relevance and knowledge retention ratio for each year group. There were increasingly positive correlations between the items that were answered correctly and their perceived relevance from Years 2 to 5, implying that items that were rated as clinically relevant were more likely to be answered correctly by the senior students than by the junior students (Figure 3).

## Discussion

This study highlights the increasingly positive correlation between perceived clinical relevance and the retention of basic science knowledge with progression to more senior years.

### What are the demographic factors that influence retention of basic science knowledge?

On the whole, in our study, retention of basic science knowledge was significantly affected by year of study, gender and student origin. The marked decline in retention of basic medical sciences with increasing student seniority has been well documented in the literature [4,7,8]. However, we observed the opposite trend amongst our students. Interestingly, the lowest scores were attributed to the Year 2 students who, in theory, have had the most recent exposure to the basic science content material. Given the intensity and overload of medical curricula, junior students often postpone studying until just before exams [27,28]. The fact that this study was conducted prior to students’ preparation for exams may have accounted for the lower performance in Year 2 and could be a reflection of little or no engagement with the learnt content. The generally better *en bloc* performance of Years 3–5 students may be a reflection of on-going teaching activities and placements reinforcing the clinical application of basic science knowledge. This is supported by evidence that prolonged contact with a domain is the greatest determinant of long term retention of knowledge [29]. Longitudinal testing of individual year-cohorts may strengthen this hypothesis.

The observed gender difference in anatomy, where males performed significantly better than females echoes the findings of Snelling et al. [30], who reported that females tend to react more adversely to cadaveric dissections. This may have reduced their level of engagement with the subject. It was also noted that generally, students performed poorly in anatomy despite the questions being similar in difficulty to those of other disciplines. It is likely that the reason for this is multifactorial in nature; one such factor may be the lower relative perceived relevance of the subject. Another reason may be that in learning anatomy, the students might have focused on memorisation at the expense of understanding [31]. Furthermore, the observed interactions indicate that there are complex and intricately interwoven relationships between demographic factors and retention of basic science knowledge. Exploratory studies would be required to further investigate the reasons for these findings.

International students had significantly lower scores than their domestic counterparts, particularly in biochemistry and pharmacology. This supports existing literature and suggests that international students may not be as well adjusted to the self-directed learning style that requires critical thinking and application of knowledge [32]. Better performance by students involved in tutoring, though not significant, points to the positive impact of tutoring on increased retention of information [33].

Innovative educational interventions may be helpful to assist those student categories (such as females in anatomy and international students) who seem to have retained lesser basic science knowledge [34-36].

### Does perceived clinical relevance of basic science materials relate to retention of knowledge?

Traditionally, there has been a disparity between learnt basic sciences and clinical sciences, where complete mastery of the former was not necessarily essential to enable the latter [5]. Echoing the reports of Custers [4], D’Eon [8] and Ling et al. [20], our findings indicate that as students progress through their medical course, they become more critical of the clinical relevance of basic science subjects, such as biochemistry and anatomy. Allied with this finding, they tend to have least retained knowledge in these disciplines. The widely-held perception that anatomy is regarded by educationalists as a difficult subject [37] may have been the reason for the observed low performance in anatomy and the subsequent low relevance ratings in this study. This may be overcome by using contemporary educational strategies that foster the development of personal framework of understanding [35] and also emphasise the relevance and clinical application of anatomy.

In spite of the tendency to lower performance of Year 5 students in comparison to their juniors (Years 3 and 4), their ratings of the clinical relevance of the questions were better correlated with their retention of knowledge. This finding confirms that perceived relevance of a subject matter fosters retention of knowledge [10,11]. The fact that their perception of clinical relevance was the most accurately correlated with retention of knowledge suggests that the senior students were able to make more precise judgements than junior students about the clinical relevance of basic science questions [12]. Too much of seemingly irrelevant material in a curriculum encourages surface learning [38]. Therefore, course evaluation by graduating students, which includes feedback about clinical relevance of biomedical science teaching, would be valuable. This outcomes-based approach, together with strategies to increase student awareness of clinical relevance, will further foster the integration of basic sciences with clinical sciences and enhance application and retention of acquired knowledge for lifelong learning. The reasons why clinical relevance is an important factor in promoting knowledge retention is not addressed in this study. It is likely however, that students would rate this information to be of greater significance, and thus rehearse, reinforce and use it more often. Knowledge that is not of use or clinically irrelevant becomes inert and inaccessible [39].

Further studies could also explore the performance and perceptions of new medical graduates (PGY1). This will provide insight into whether further experience in the workplace reinforces relevant information presented early in medical school or not. In addition, inclusion of Year 1 students could serve as a useful control group for future studies. Exploratory studies could help elucidate reasons for the differences in perceptions of relevance of the different basic science subjects and possibly result in targeted educational strategies that support reinforcement and progressive retention of gained knowledge.

### Limitations of the study

Generalisability of the findings from this study may be limited by several factors. Our definition of clinical relevance may have unduly constrained the participants’ perception of clinical applicability, although care was taken to explain the term to the participants at the start of the assessment. Furthermore, the applicability of the results of this study may have been restricted by the use of voluntary participation and the fact that it was a low stakes examination. However, comparison of performance on regular in-course examinations indicated that participants were representative of their respective cohorts. This limitation was also mitigated by the administration of the assessment prior to students’ preparation for their in-course examination. This allowed for better elucidation of retained basic science knowledge.

The study could have also benefited from the use of more items per discipline to allow for greater breadth of content within each discipline, but this would have increased the duration of the examination and possibly discouraged participants. Further, due to the random selection of validated questions within each discipline, some questions may not have had a clinical stem, which could have biased students’ perception of clinical relevance. However, this is unlikely to have been skewed with respect to any particular basic science discipline.

Although we have inferred perceived clinical relevance, as measured by our survey, to have causal influence over knowledge retention of basic science items, it is conceivable that in some instances, the direction of causality may in fact be reversed. Students may declare relevance for facts that they know well or have previously rehearsed, although the degree of such rehearsal is unknown and likely to be variable. While this is worthy of further enquiry, the present study cannot address it in any more detail. Obtaining independent ratings of clinical relevance may be one way of resolving the issue.

Finally, while it could be postulated that future career aspirations may influence performance and perceptions of relevance with respect to certain basic sciences, this could not be confirmed by the study, which was likely due to the fact that the questions asked were not sufficiently rigorous to elucidate adequately interpretable responses. This should be the focus of ongoing work.

## Conclusions

The results of this study suggest that a CBL curriculum can provide a platform for continued formal integration of the basic sciences with clinical sciences, although further research comparing results in CBL and non-CBL settings is required for the generalisability of our findings. Understanding the clinical relevance of basic science knowledge may directly or indirectly be an important factor in the retention of basic science knowledge and its ultimate application in the clinical context. However, a proactive reinforcement and systematic review of basic sciences in the later years of undergraduate medical education may also be needed to ensure knowledge retention.

## Competing interests

The authors declare that they have no competing interests.

## Authors’ contributions

BMA conceived the study; BMA and AYSL collected and analysed the data. All authors advised on research methodology, data analysis and interpretation. All authors critically revised the manuscript for intellectual content and approved the final manuscript.

## Pre-publication history

The pre-publication history for this paper can be accessed here:

http://www.biomedcentral.com/1472-6920/13/139/prepub

## Supplementary Material

Additional file 1 Factors that determine knowledge retention*. (*for each trait, means bearing different superscripts within the same column are significantly different (p < 0.01). Click here for file

## References

[B1] Kappagoda A Training doctors - too long in the cellar? Med J Aust 2012 196 8 489 10.5694/mja12.c0507 22571296

[B2] Bonaminio G The challenge of integrating the basic medical sciences Basic SciEduc 1998 8 1&2 13 15

[B3] Bligh J Learning about science is still important Med Educ 2003 37 11 944 945 10.1046/j.1365-2923.2003.01703.x 14629402

[B4] Custers EJFM Long-term retention of basic science knowledge: a review study Adv Health Sci Educ Theory Pract 2010 15 1 109 128 10.1007/s10459-008-9101-y 18274876

[B5] Lazic E Dujmovic J Hren D Retention of basic sciences knowledge at clinical years of medical curriculum Croat Med J 2006 47 6 882 887 17167861 PMC2080481

[B6] Norman G The essential role of basic science in medical education: the perspective from psychology Clin Invest Med 2000 23 1 47 51 10782317

[B7] Gonnella JS Hojat M Erdmann JB Veloski JJ Assessment measures in medical school, residency, and practice: the connections 1993 New York: Springer

[B8] D’Eon M Knowledge loss of medical students on first year basic science courses at the University of Saskatchewan BMC Med Educ 2006 6 1 5 10.1186/1472-6920-6-5 16412241 PMC1397826

[B9] Ling Y Swanson DB Holtzman K Bucak SD Retention of basic science information by senior medical students Acad Med 2008 83 10 S82 S85 18820508 10.1097/ACM.0b013e318183e2fc

[B10] Harris JA Heneghan HC McKay DW The rating of pre‒clerkship examination questions by postgraduate medical students: an assessment of quality and relevancy to medical practice Med Educ 2003 37 2 105 109 10.1046/j.1365-2923.2003.01403.x 12558880

[B11] Entwistle NJ Influences on the quality of student learning—implications for medical education S Afr Med J 1992 81 2 596 605 1621169

[B12] What makes for success in medical education? Synthesis report http://www.innovation.gov.au/highereducation/ResourcesAndPublications/Documents/HEReports/SynthesisReport.pdf

[B13] Grebennikov L Skaines I University of western Sydney students at risk: profile and opportunities or change J Institut Res 2009 14 1 58 70 10.1007/s10310-008-0105-5

[B14] McKenzie K Schweitzer R Who succeeds at university? Factors predicting academic performance in first year Australian university students Higher Educ Res Dev 2001 20 21 33 10.1080/07924360120043621

[B15] Smits PB Verbeek JH Nauta MC Ten Cate TJ Metz JC van Dijk FJ Factors predictive of successful learning in postgraduate medical education Med Educ 2004 38 7 758 766 10.1111/j.1365-2929.2004.01846.x 15200400

[B16] Schank RC Schank RC, Langer E Goal-based scenarios Beliefs, reasoning and decision making: psycho-logic in honour of Bob Abelson 1994 Hillsdale, NJ: Lawrence Erlbaum Associates 1 32

[B17] Srinivasan M Wilkes M Stevenson F Nguyen T Slavin S Comparing problem-based learning with case-based learning: effects of a major curricular shift at two institutions Acad Med 2007 82 1 74 82 10.1097/01.ACM.0000249963.93776.aa 17198294

[B18] Thistlethwaite JE Davies D Ekeocha S Kidd JM MacDougall C Matthews P Purkis J Clay D The effectiveness of case-based learning in health professional education. A BEME systematic review: BEME Guide No. 23 Med Teach 2012 34 6 e421 444 10.3109/0142159X.2012.680939 22578051

[B19] Tärnvik A Revival of the case method: a way to retain student-centred learning in a post-PBL era Med Teach 2007 29 1 32 36 10.1080/01421590601039968 17538830

[B20] Irby DM Three exemplary models of case-based teaching Acad Med 1994 69 12 947 953 10.1097/00001888-199412000-00003 7999181

[B21] Malau-Aduli BS Zimitat C Peer review improves the quality of MCQ examinations Assess Eval High Educ: An Int J 2011 37 8 919 931 ISSN 1469-297X doi:10.1080/02602938.2011.586991

[B22] Hazlett C Yip S Nicholls J Au W Tan J IDEAL-HKTM PC Users’ guide: instructors & system administrators 2003 Hong Kong: Condor Production Ltd

[B23] Faul F Erdfelder E Lang AG Buchner A G* power 3: a flexible statistical power analysis program for the social, behavioral, and biomedical sciences Behav Res Methods 2007 39 2 175 191 10.3758/BF03193146 17695343

[B24] SAS Statistical Analysis System institute North Carolina USA 2002 Cary, NC: SAS Institute Inc version 9.1

[B25] Cohen J Statistical power analysis for the behavioural scienecs 1988 2 Hillsdale, NJ: Lawrence Erlbaum Associates

[B26] Precht D Hazlett C Yip S Nicholls J Item analysis User’s guide 2003 Hong Kong: International Database for Enhanced Assessments and Learning

[B27] Pychyl TA Morin RW Salmon BR Procrastination and the planning fallacy: an examination of the study habits of university students Soc Behav Personal 2000 15 5; SPI 135 152

[B28] Solomon LJ Rothblum ED Academic procrastination: frequency and cognitive-behavioral correlates J Couns Psychol 1984 31 4 503

[B29] Conway MA Cohe G Stanhope N On the very long-term retention of knowledge acquired through formal education: twelve years of cognitive psychology J Exp Psych:Gen 1991 120 4 395 409

[B30] Snelling J Sahai A Ellis H Attitudes of medical and dental students to dissection Clin Anat 2003 16 2 165 172 10.1002/ca.10113 12589673

[B31] Miller SA Perrotti W Silverthorn DU Dalley AF Rarey KE From college to clinic: reasoning over memorisation is key for understanding anatomy Anat Record 2002 269 69 80 10.1002/ar.10071 12001213

[B32] Malau-Aduli BS Exploring the experiences and coping strategies of international medical students BMC Med Educ 2011 11 40 10.1186/1472-6920-11-40 21702988 PMC3141796

[B33] Amorosa JMH Mellman LA Graham MJ Medical students as teachers: how preclinical teaching opportunities can create an early awareness of the role of physician as teacher Med Teach 2011 33 2 137 144 10.3109/0142159X.2010.531154 21275543

[B34] Rondon S Sassi FC de Andrade CR F Computer game-based and traditional learning method: a comparison regarding students’ knowledge retention BMC Med Educ 2013 13 30 10.1186/1472-6920-13-30 23442203 PMC3586342

[B35] Pandey P Zimitat C Medical students’ learning of anatomy:memorisation, understanding and visualisation Med Educ 2007 41 1 7 14 10.1111/j.1365-2929.2006.02643.x 17209887

[B36] Storr EF International Medical Students: factors that enhance and inhibit learning Thesis, Master of Health Sciences 2013 University of Otago Retrieved from http://hdl.handle.net/10523/3904

[B37] Turney B Anatomy in a modern medical curriculum Ann R Coll Surg Engl 2007 89 2 104 10.1308/003588407X168244 17346399 PMC1964553

[B38] Ramsden P Learning to teach in higher education 2003 2 London: Routledge Falmer

[B39] Ten Cate O Snell L Mann K Vermunt J Orienting teaching toward the learning process Acad Med 2004 79 3 219 228 10.1097/00001888-200403000-00005 14985194

