# Supplementary material for: Retention of knowledge and perceived relevance of basic sciences in an integrated case-based learning (CBL) curriculum
Source: BMC Med Educ. 2013 Oct 8;13:139. doi: 10.1186/1472-6920-13-139 (PMC3851808; doi:10.1186/1472-6920-13-139)
Supplement: Additional file 1 — Factors that determine knowledge retention*. (*for each trait, means bearing different superscripts within the same column are significantly different (p < 0.01). [file 1472-6920-13-139-S1.pdf]

Additional File 1: Factors that determine knowledge retention*

Trait	No of Obs	Biochemistry	Anatomy	Pathology	Pharmacology	Physiology	Overall Score
**Year of Study**							
2	44	50.23±17.72[b]	44.77±22.36	51.59±14.46[b]	54.77±21.18	47.95±18.50	49.86±13.08[b]
3	71	65.21±20.27[a]	50.70±20.52	59.72±15.58[a]	55.21±17.72	49.15±17.55	56.00±11.70[a]
4	62	61.61±20.34[a]	47.26±21.67	64.35±17.52[a]	47.90±18.57	56.29±15.60	55.48±12.64[a]
5	55	62.73±17.15[a]	41.45±18.20	60.55±14.20[a]	53.27±16.34	51.27±18.56	53.85±11.37[ab]
**Origin**							
Domestic	184	63.10±19.18[a]	47.34±21.35	60.54±15.85	55.11±17.89[a]	52.12±17.72	55.64±11.98[a]
International	48	52.08±19.57[b]	43.13±18.58	56.04±16.73	43.54±17.92[b]	48.33±17.30	48.63±11.94[b]
**Sex**							
Male	102	60.49±22.09	52.06±20.60[a]	60.00±15.61	53.14±17.69	53.82±18.67	55.90±12.77
Female	130	61.08±17.75	42.08±20.03[b]	59.31±16.53	52.38±19.12	49.38±16.65	52.85±11.76
**Age-Group**							
18-21 years	88	60.00±1994	50.23±22.07	57.27±16.59	56.48±19.00	50.45±18.06	54.89±13.04
22-25 years	126	60.63±19.59	43.17±19.58	60.63±16.14	50.16±18.46	51.35±17.22	53.19±12.05
>25 years	18	66.11±20.04	51.11±20.26	63.89±11.95	52.22±12.63	55.56±19.17	57.78±9.40
**Tutoring**							
Yes	50	66.00±17.96	45.60±22.42	61.20±14.93	55.00±18.32	56.20±16.65	56.80±12.74
No	182	59.40±20.00	46.70±20.44	59.18±16.42	52.09±18.51	50.00±17.75	53.47±12.09
**Total**	232	60.81±19.73	46.46±20.83	59.61±16.09	52.71±18.46	51.33±17.66	54.18±12.27

*For each trait, means bearing different superscripts within the same column are significantly different ($p<0.01$)
